# Bamboo-Fiber-Reinforced Thermoset and Thermoplastic Polymer Composites: A Review of Properties, Fabrication, and Potential Applications

**DOI:** 10.3390/polym14071387

**Published:** 2022-03-29

**Authors:** A.M. Radzi, Sheikh Ahmad Zaki, Mohamad Zaki Hassan, R.A. Ilyas, Khairur Rijal Jamaludin, Mohd Yusof Md Daud, Sa’ardin Abd Aziz

**Affiliations:** 1 Malaysia-Japan International Institute of Technology, Universiti Teknologi Malaysia, Jalan Sultan Yahya Petra, Kuala Lumpur 54100, Malaysia; mohd.radzi@utm.my; 2 Razak Faculty of Technology and Informatics, Universiti Teknologi Malaysia, Jalan Sultan Yahya Petra, Kampung Datuk Keramat, Kuala Lumpur 54100, Malaysia; khairur.kl@utm.my (K.R.J.); yusof.kl@utm.my (M.Y.M.D.); saa.kl@utm.my (S.A.A.); 3 School of Chemical and Energy Engineering, Faculty of Engineering, Universiti Teknologi Malaysia, Johor Bahru 81310, Malaysia; ahmadilyas@utm.my; 4 Centre for Advance Composite Materials (CACM), Universiti Teknologi Malaysia, Johor Bahru 81310, Malaysia

**Keywords:** bamboo fibers, hybrid, composites, thermoplastic, thermoset, mechanical, thermal

## Abstract

Natural-fiber-reinforced composites, especially bamboo, are an alternative material to compete with conventional materials. Their environmentally friendly, renewable, low-cost, low-density, non-toxic, and fully biodegradable properties are concerning for researchers because of their advantages over synthetic polymers. This comprehensive review presents the results of work on bamboo fiber composites with special reference to bamboo types, thermoplastic and thermoset polymers matrices, hybrid composites, and their applications. In addition, several studies prove that these properties are very good and efficient in various applications. However, in the development of composite technology, bamboo fiber has certain constraints, especially in moisture conditions. Moisture is one of the factors that reduces the potential of bamboo fiber and makes it a critical issue in the manufacturing industry. Therefore, various efforts have been made to ensure that these properties are not affected by moisture by treating the surface fibers using chemical treatments.

## 1. Introduction

Natural-fiber-reinforced polymer composite (NFP) is a composite material that is combined with natural fiber and polymer. Composite structures are generally a combination of two or more materials at the macroscopic level and both are insoluble. The natural fiber is a reinforcement material embedded in a polymer (matrix) where the polymer has two types of classes, namely thermoplastic and thermoset [1,2]. Over the past few decades, what can be seen around us is the production of a wide range of products that use natural-fiber-reinforced polymer composites. This composite is one of the alternatives to produce environmentally friendly materials by combining polymers and natural fibers for use in various products applications [3,4]. The use of these natural fibers has a high impact on the manufacturing industry as these materials are readily available, low-cost, and easy to design and increase productivity [5].

Nowadays, the use of petroleum-based plastics in human daily activities is increasing [6]. With the increasing use of plastics among us, plastic disposal has become a major issue of environmental pollution, and, in addition, limited fossil fuel resources make many researchers look for alternative methods to reduce the use of petroleum-based matrices [7,8,9,10]. Therefore, the solution to this problem is to combine both materials using polymers and natural fibers. Furthermore, the use of natural resources material is to reduce the abundance of waste and prevent open burning by farmers leading to air pollution [11,12,13]. Thus, the nature of awareness of environmental issues to the world community nowadays is increasing. This increase in awareness occurs when global warming occurs in the world, and when loss of biodiversity and garbage disposal problems occur around them. Therefore, various activities have been held among them, such as recycling programs, segregation of waste by type, and use of natural product materials [14,15,16].

Natural-fiber-reinforced composite fibers are one of the alternatives to reduce environmental problems, and there is a need to further enhance the capabilities of this green technology [17,18,19,20]. Natural fiber composites are in high demand in manufacturing industries, such as transmission tower, automotive, construction, aerospace, as well as furniture and packaging [21,22,23,24,25,26]. One of the natural resources emphasized is bamboo trees.

Nowadays, many researchers have studied bamboo to be extracted into fiber and made this fiber a reinforced material in the polymer matrix. The physical, mechanical, and thermal properties of bamboo-based composites have been investigated in a few previous review studies, as summarized in Table 1. The selection of bamboo as a reinforcement is due to its good mechanical and thermal properties, extraction and fiber treatment, low cost, environment friendly nature, and ability to be used as a product in the industry [27,28]. Bamboo fiber is identified to have strength and stiffness, and it contains microfibrillar angles and thick cell walls that are considered nature glass fiber [29,30,31].

In addition, the content of bamboo has 60% cellulose and lignin, and the microfibril angle is between 2° and 10°. Therefore, polymer composites combined with bamboo fiber can compete with conventional fibers and also have the potential to be used as a product in the manufacturing industry sectors, such as automotive parts, furniture, building, and packaging.

This review aims to focus on the trends of the physical, mechanical, and thermal properties of bamboo-fiber-reinforced thermoset and thermoplastic polymer, hybrid composites, and their application. In addition, there is comprehensive research on bamboo in terms of engineering, type, cultivation, and fiber capability in the manufacturing industry.

### 1.1. Natural Fibers

Natural fiber can be found in Southeast Asia (Malaysia, Indonesia, and Thailand) and South America [39,40,41,42]. According to Taj [43], natural fiber production worldwide is more than 25 million tons per year. Table 2 shows the annual production of natural fiber.

Natural fiber sources are increasingly gaining attention for use as fiber-reinforced polymer composites, such as polylactic acid [45], chitosan [46], polycaprolactone [47], and thermoplastic starch [48,49,50,51,52,53]. These natural fibers include leaf fibers, seeds, wood, straw, and grass. Plant fibers are alternative materials used as fillers in the polymer matrix and are easily available, such as bamboo, rice straw, sugar palm fiber, kenaf, roselle, and pineapple [23,24,25]. Most of these fibers have a chemical composition structure consisting of lignin, cellulose and hemicellulose, wax, as well as inorganic and water-soluble compounds. Table 3 and Table 4 show the chemical composition, as well as the mechanical and physical properties of the natural and E-glass fibers. Additionally, the effects of using this material has a positive impact because it is low-cost, reusable, biodegradable, environmentally friendly, and sustainable [54,55,56,57].

A natural resource that is easily available in Malaysia is the Bamboo plant. This bamboo can produce fiber to be used as one of the fiber materials as the reinforcement polymer matrix. From time immemorial, bamboo is easy to use and can be applied to products (living tools) because of its strength. Furthermore, bamboo is a tree that is easy to find and grow in Asia and the United States [40]. The use of bamboo has grown as materialist is used to produce various products and is a source of economic livelihood for some communities. This bamboo cultivation takes several months to reach maturity or can be used for various applications [63,64].

### 1.2. Bamboo Plant

**Figure 1:**
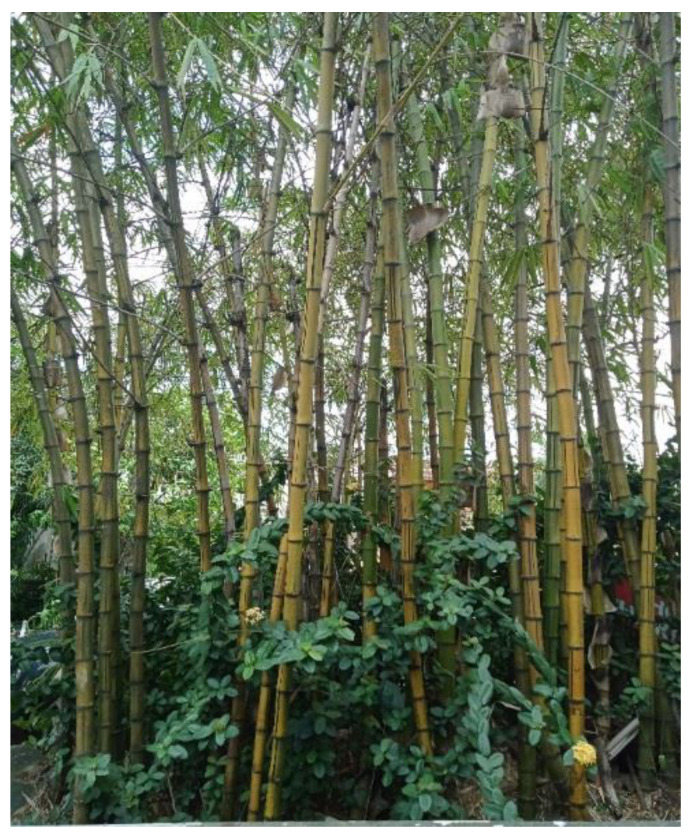
Bamboo tree.

Bamboo is from the family ‘*Gramineae*’ group where it belongs to the types of grasses, such as weeds, rice, corn, and sugarcane. Figure 1 shows the bamboo plant. More precisely, it also belongs under the *Bambusoideae* subfamily [37,64]. In addition, bamboo contains genus, such as *Bambusa*, *Dendrocalamus*, *Dinochloa*, *Gigantochloa*, *Schizostachyum*, *Holttumochloa*, *Kinabaluchloa*, *Maclurochloa*, *Melocanna*, *Chusquea*, *Phyllostachys*, *Soejatmie*, *Sphaerobambos*, and *Thyrsostachys*, etc., in this sub-family [65].

Bamboo cultivation is easy and fast, and there are large quantities of it too. Bamboo is fertile in moist areas because it uses a lot of water as a growth agent. Therefore, in the Malaysian peninsular with a humid climate, this bamboo is easily found in areas near rivers, in forests, in bushes, and in derelict areas. Areas such as swamps and muddy areas are not suitable for bamboo growers because of their genetic factors that cannot be submerged even though the bamboo needs water to grow.

Statistics have been prepared, showing that about 200 species of bamboo are planted or grown wild in Southeast Asia, namely Malaysia, Myanmar, Indonesia, and Papua New Guinea [46,47]. In Peninsular Malaysia, there are approximately 63 types of bamboo that are systematically planted or grown wild [66]. From the 63 bamboo species, there are only 13 types that can be used to make various products commercially or traditionally. Nowadays, the uses and products of bamboo are in line with modern technology in the new millennium. The production of products such as laminated board, particleboard, and ply bamboo, as well as types of *B. vulgaris* (*Bambusa vulgaris*) and *G. scortechinii* (*Gigantocchloa scortechinii*), is suitable compared to other types of bamboo. Therefore, an evaluative comprehensive study needs to be conducted on all types of bamboo in various forms of final products [66,67].

### 1.3. The Anatomy of the Bamboo Tree

**Figure 2:**
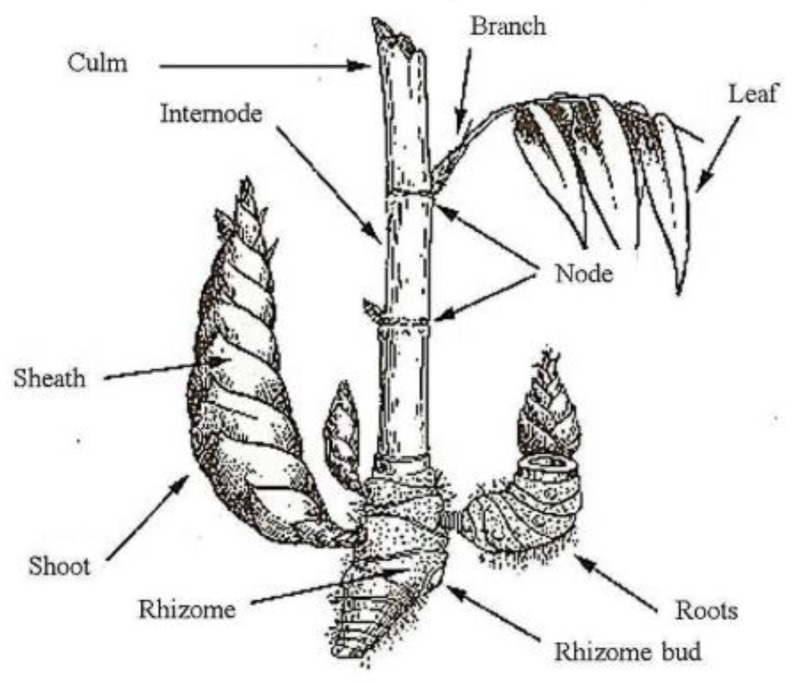
Bamboo anatomy [36].

**Figure 3:**
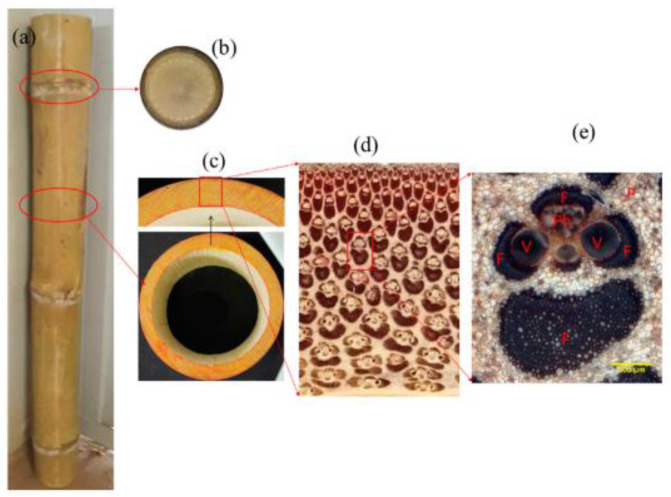
Morphological characteristics of bamboo: (**a**) bamboo culm; (**b**) node diaphragm; (**c**) internode; (**d**) culm wall; and (**e**) vascular bundle [69].

The anatomy of the bamboo tree is the leaves, stems, roots, rhizome, branches, and shoots. There are several species of bamboo, such as *Phyllostachys Elegans* and *Phyllostachys edulis* ‘*Moso*’, which produce flowers spontaneously [36]. Flowering phenomena (*sporadic*) occur due to the surrounding environment rather than genetics. In general, this bamboo tree grows longitudinally and has no lateral compared to other trees. Typically, these bamboo structures have intermittently covered hollow stems called ‘nodes’. Figure 2 and Figure 3 show the bamboo anatomy and morphological structure of bamboo culm. The contents of this bamboo consist of parenchyma, fiber, vessels, and a sieve tube [68,69]. Each bamboo structure consists of 50% parenchyma, 40% fibers, and 10% vessels and sieve tubes [70,71]. The fiber content of bamboo, one-third of the bamboo wall, and the upper part of the stem have the highest fiber content when compared to the other parts of the bamboo. In addition, bamboo also contains cellulose and pectin (wax coating) on the outer surface of the bamboo stem. In fact, silica content is also found in bamboo stems where it is more concentrated in the peripheral parts of the culm.

## 2. Bamboo Plantation in Malaysia

The bamboo tree is a multi-purpose plant that is often used by the rural population in Malaysia. Bamboo is also a source of food, especially in the shoots, and the culm part is applied to multi-purpose products, such as household appliances, bridges, baskets, sticks, skewers, handicrafts, and others. This bamboo easily grows in logging areas, hillsides, and river banks. These bamboo trees easily compete or mix with other tree species in the forest. The expected bamboo plantation exceeds 421 ha which covers 6.9% of the forest in peninsular Malaysia [72,73]. In Malaysia, only 12 types of bamboo are used commercially even though the bamboos found here are many and abandoned. Bamboo types, including Bambusa blumeana (*thorny bamboo*), B. vulgaris (*aur*/*oil bamboo*), B. heterostachya (*pole bamboo*), Gigantochloa scortechinii (*semantan bamboo*), G. thoii (*bamboo betting*), G. ligulata (*dense bamboo*), G. wrayi (*beti bamboo*), and Schizostachyum brachycladum (*lemang bamboo*), are easily available and often used commercially in Malaysia [27,66,74]. Nowadays, the use of bamboo has expanded according to the latest technology. With recent advancements in technology, the use of bamboo is focused on environmentally friendly and low-cost materials that can be made into composite products with stronger properties than single bamboo.

### 2.1. Bamboo-Based Polymer Composites

Composites are a growing material to be applied to a variety of products applications. The combination of natural fibers and polymer matrix can produce an excellent product according to the desired standards. In general, the selection of natural resources is more environmentally friendly compared to the selection of conventional fibers (glass and carbon fiber). In fact, these resources provide a positive impact and provide advantages equivalent to conventional materials, such as lower density, recyclability, and compatibility of both materials [75,76,77]. This combination is focused on two types of matrix polymers, namely thermoplastic and thermoset. Natural fiber, such as bamboo fiber, is one of the materials used to be combined or reinforced with polymer matrix. Several researchers have conducted studies on bamboo-fiber-reinforced polymer composites, namely on their mechanical, physical, and thermal properties. Bamboo fiber has high-gravity-specific properties when compared to wood used in manufacturing applications. Moreover, it has high mechanical properties and is comparable to wood given that it can make a significant contribution to the composite material [36,70,78]. Table 5 shows the mechanical properties of bamboo species. The mechanical properties possessed by bamboo are better than those of wood. The use of bamboo materials for various applications is very suitable, such as oriented structural boards, which are boards that can bear the unidirectional load.

### 2.2. Bamboo-Reinforced Polymer Thermoplastic Composites

Many researchers have studied to see the potential of bamboo fiber for solving various major problems such as environmental problems, recyclability, wettability, liability, and affordability on bamboo fibers. According to Aji et al. [79] and Torress and Diaz [80], this study intends to reach an oriented conclusion based on the composite for product application. These properties are due to the fiber distribution, fiber age, and method of cultivation, and fiber extraction can contribute to the effect of the composite properties [81]. Therefore, fiber distribution or alignment factors are also significant as can influence the properties of the composite. Table 6 shows the bamboo-reinforced polymer thermoplastic composites and testing method.

Recently, the use of natural fibers has gained interest from a variety of industries, such as automotive, textile, and furniture industries, where natural fibers have high strength and are environmentally friendly. Natural fibers, such as bamboo fibers, are also considered to have good mechanical strength. The mechanical properties of bamboo composites are usually tested, for example, through tensile, flexural, and impact tests. Tensile testing is the force exerted on the maximum level of the composite bamboo to test its withstanding ability before it breaks. Typically, these test samples are dumble in shape and follow the standards specifications. The bending strength is used to test the ability of the bamboo composite to push the shape under applied pressure, while impact strength is used to measure the absorption force and energy loss when a force is applied suddenly on the bamboo composites.

Yeh and Yang [87] investigated the effect of different waste bamboo-fiber-reinforced PP composites. There are four types of bamboo waste, namely Makino bamboo (*Phyllostachys makinoi*), Moso bamboo (*Phyllostachys pubescens*), Ma bamboo (*Dendrocalamus lactiferous*), and Thorny bamboo (*Bambusa stenostachya*), which are used as reinforcing PP composites on tensile and flexural properties. The results indicate that Makino bamboo impacts the tensile and modulus properties. This happened because Makino bamboo has high crystallinity and high lignin content when compared to other bamboo wastes. In addition, the good bonding between the fiber and matrix can improve the mechanical properties [88,89,90,91]. For the flexural properties, Ma bamboo is the highest modulus of rupture and modulus of elasticity. These mechanical tests are influenced by intrinsic elements of stiffness and chemical composition (cellulose and lignin). Yeh and Yang [87] and Jarvis [92] agreed that the strength present was due to cellulose and lignin being naturally present in bamboo waste. Among other natural fibers, bamboo fiber shows good potential and the combination in polymer composites is also great. Table 7 shows the mechanical and physical properties of bamboo-reinforced thermoplastic polymers. The combination of bamboo fiber with thermoplastics, such as high-density polyethylene (HDPE), low-density polyethylene (LDPE), polypropylene (PP), polystyrene, and polylactic acid (PLA) with various sizes and uniformity, and fiber loading bamboo fibers shows an improvement in their mechanical properties up to optimum value. Therefore, the characteristics of size, uniformity, and fiber content are closely related to good mechanical properties of the bamboo composite.

The execution of bamboo-fortified polymer composites is more often than not measured by their physical and thermal properties, such as water assimilation, pliability, thermogravimetric analysis (TGA), differential scanning calorimetry (DSC), and energetic mechanical investigation (DMA). TGA on composites can determine the reactions and physical changes in the composite with the mass loss. The thermal properties of bamboo composites have also been proven from previous studies where the effect of heat also influences the behavior of bamboo and composites. Ren et al. [85] investigated the effect of bamboo pulp fiber-reinforced PE composites with different fiber loading. TGA thermal was performed on bamboo and PE and was supported by derivative thermogravimetric (DTG) analysis. The TGA and DTG showed four-phase degradation of the bamboo-reinforced PE composites, i.e., loss of moisture content, degradation of hemicellulose, degradation of cellulose/lignin, and residual ash. Additionally, the thermal stability increased when the bamboo content increased, as compared to neat PE and bamboo flour. Table 8 shows the TGA results for bamboo–HDPE composites and neat HDPE.

Sanjay et al. [86] studied the effects of fiber loading on the mechanical and thermal properties of bamboo-reinforced PP composites. The different prepared contents were 10 to 60 vol%, using the hot compression method. Heat deflection temperature (HDT) was carried out for thermal properties. From the result, the HDT shows increased by increasing the bamboo contents compared with neat PP. This increase occurs following the increase in limited polymer chain movement due to the high bamboo content. In addition, the strength of the interface between the matrix and the bamboo is one of the factors where this increase occurs [86,103].

### 2.3. Bamboo-Reinforced Thermosetting Polymer Composites

Thermoset-type polymer materials are often used in a variety of applications in the manufacturing industry, such as adhesives, coatings, insulation, and mold compounds. Commonly used polymers are vinyl ester (VE), epoxy, phenolic, polyimide, and polyester [104]. The use of thermoset polymer is due to its unique properties where it has dimensional stability, creep resistance, chemical resistance, and stiffness [22,105,106]. In fact, its structure also cannot be changed to other forms and cannot be recycled compared with thermoplastic materials. Table 9 presents a list of different types of thermoset polymer with their mechanical and density properties. These polymers are often combined with natural or synthetic fibers to obtain optimum properties.

This combination will form fiber-reinforced polymer composites used for a variety of applications such as automotive parts, furniture, and construction and protection materials. Table 10 shows gathered information on bamboo-reinforced thermoset polymer and testing methods.

To date, the study of bamboo-reinforced thermoset composites is growing and interesting. The reason why researchers are turning to a combination of bamboo and thermoset is because of its more environmentally friendly properties and easy-to-find source materials compared to conventional materials that require a certain cost to produce. Several researchers have conducted studies on the use of bamboo-reinforced thermoset polymer composites, as shown in Table 10. Overall, researchers have stated that the use of natural fibers, especially bamboo, has shown good performance from a technical point when compared to hardwood. These properties of bamboo are generally considered to be a flexible material, but are physically stiff and comparable to hardwood. Therefore, the whole bamboo section can be produced a variety of product applications.

Mechanical properties of bamboo-based and bamboo-reinforced thermoset polymer composites are influenced by several factors similar to thermoplastic matrix, namely fiber content, distribution, interface adhesion, and fiber aspect ratio. Rao et al. [118] studied the effects of water uptake and the mechanical properties of outdoor bamboo-fiber-reinforced with different concentration PF composites (10–25 wt.%). The method used in this experiment is PF-impregnated with different concentrations into bamboo fiber for 4–8 min and dried at room temperature. Then, the bamboo is through a hot pressing process at a temperature of 150 °C for 0.5 min at a pressure of 3.5–7 Mpa, respectively. The result of the mechanical (bending and compressive) test shows increased performance when increasing the matrix at a 10–20 wt.% concentration. Bamboo has different density values when pressure is applied to it during the fabricate process and when the structure of bamboo and the walls of the cells are deformed (wrinkled) and crushed. Due to this effect, the resin will penetrate into the damaged bamboo structure, vessel, and lumina, and will react with the interface between the bamboo and the matrix to form the inner wall to improve the properties of the bamboo composite. The stiffness of the polymer matrix is lower compared to bamboo fibers; this facilitates penetration, redistribution, and solidification on bamboo fiber composites. This formation phenomenon is one of the factors which contribute to the improvements in the mechanical properties of bamboo composites [118,119,120]. In addition, the water absorption test has shown a gradual increase in absorption with increasing matrix into the composite. The absorption process does not occur significantly from 20 wt.% up to 25 wt.% matrix concentration. This indicates that the hydrophobic nature of the PF has helped to reduce the absorption of composites [103,104]. Therefore, the authors argue that increasing the concentration of matrix on bamboo has a positive effect and has the potential to be applied to various products. Table 11 shows bamboo-fiber-reinforced thermoset polymer composites on their properties. From previous research, bamboo fiber is a raw material used in the manufacturing industry that can be made and produced in a variety of products as a reinforcing material in the thermoset. Similar to thermoplastic polymer, thermosets, such as epoxy, phenolic, polyester, etc., have characteristics of size, uniformity, and fiber contents which are closely related to mechanical properties (tensile, flexural, and impact strength) of the bamboo composite.

The thermal properties of the bamboo/thermoset polymer are also performed to determine the stability of the composites. This test also helps to determine thermal stability. Huang et al. [112] studied the effects of different untreated and treated fiber lengths and fiber content on the mechanical and thermal properties of bamboo-reinforced epoxy composites. In this study, a comparison on mechanical and thermal properties was conducted between untreated fiber and fiber treated with sodium hydroxide (NaOH) solution to measure the ability of these bamboo fiber composites. From the mechanical results, treatment and non-treatment effects showed improvement with increasing fiber content and fiber length. In the bending test, the opposite occurs where the effect of the treatment on the fiber shows a decrease in its properties. This effect is due to damage to the fiber during treatment, matrix cracking, fiber pull-out failure, and debonding [126,127,128]. The investigation into thermal properties composites was carried out by TGA (TGA- Q50 V20.13). The TGA was measured on untreated and treated bamboo composites and neat epoxy. The bamboo composite was treated on a 6 wt.% NaOH sample. From the result, the phenomena produced are similar to those of thermoplastic composites. There are four phases involved: loss of moisture content, degradation of hemicellulose, degradation of cellulose/lignin, and residual ash. Thermal TGA was performed on untreated and treated bamboo composites and neat epoxy and was supported by differential thermal analysis and thermogravimetric differential (DTG) analysis. During the TGA test at temperatures between 40 and 105 °C, there was a change in weight on all samples where the water was evaporated between the matrix and the fibers. According to the author, at temperature ranges of 200–330 °C, 330–356 °C, and 356–450 °C, several phases occur in which the decomposition of hemicellulose, cellulose, and lignin occurs [94,108,109]. The observations showed that untreated fiber showed a higher percentage of ash residue compared to treated and neat epoxy treatment. NaOH treatment on the fiber causes some of the lignin elements to also be affected on the fiber. The results carried out show that untreated bamboo composites have more thermal stability than NaOH treated and neat epoxy. Therefore, the improvement in chemical treatment for thermal stability depends on the temperature and immersion time, the type of treatment, and chemical concentration. The immersion time and excessive concentration have also affected the surface of the fibers as well as the thermal and mechanical properties.

### 2.4. Bamboo Fiber Hybrid Composites

The hybridization of two types of filler materials presents differences in chemical, physical, and morphological structure, which can have a positive effect on the polymer matrix. The hybrid composites (more than one fiber) can withstand high forces when they are subjected to pressure compared to a single composite [129,130]. Various techniques are used to produce hybrids composites, such as hydraulic press, hand lay-up and compression molding, twin-screw extruder, and injection molding [130]. Hybrid composites have been a concern to many researchers, and there is an aim to improve the properties of the composite. In hybrid studies, researchers have also used two materials in their study for combinations, such as natural–natural and natural–synthetic materials, in order to improve the mechanical properties of composites. Natural and synthetic fibers that are always used are kenaf, bamboo, sugar palm, rice husk, banana, coconut roselle, glass, ceramics, and carbon. One of the natural fibers that have the potential to be used as a filler is bamboo. The incorporation of bamboo with other synthetic or natural fibers increases the strength of mechanical properties and physical hybrid composites. In addition, this combination also has the potential to improve the interface bonding and uniformity of fiber dispersion. Table 12 shows the bamboo-reinforced polymer composites and preparation methods.

The mechanical properties of bamboo composites are relatively low due to less stiffness and brittle. The combination of more than two types of materials, namely from natural fiber sources or synthetic materials, can increase the mechanical and thermal properties of composites. In addition, it can reduce the water absorption on the material [60].

Sathish et al. [140] studied the effect of volume fraction on the mechanical (tensile, flexural, and impact) and physical (void content and water uptake) properties of flax and bamboo hybrid composites. In this study, all samples were fabricated with different volume fractions (0:40, 10:30, 20:20, 30:10, and 40:0) of fiber. The tensile, flexural, and impact results indicated an increase in light of the increasing volume fraction of flax on the bamboo. The combination at a ratio of 30:10 shows an excellent improvement between these two fibers where the tensile strength achieves the highest results when compared to the other ratios. From the single composite test, flax showed the highest value when compared to bamboo fiber. The combination of these two fibers shows that bamboo has the potential to have a good impact in combination with other natural and synthetic. The enhancement of mechanical properties because flax and bamboo have good interface bonding, and, besides that, flax properties have excellent modulus properties. The addition of bamboo makes the composite hybrid interact well between the fiber and the matrix. Additionally, bamboo can also infiltrate between flax fibers to reduce pores in hybrid composites. This is demonstrated by the water absorption test on hybrid composites, the test results from which show a ratio of 30:10 which can reduce water absorption. Similar studies also have been conducted by Ismail et al. [134] to determine the void content, tensile strength, and vibration properties of kenaf–bamboo hybrid composites. In the study, the ratio hybridization of kenaf and bamboo were 30:70, 50:50, and 30:70. From the tensile result, the ratio of 50:50 shows the highest value compared with another ratio hybrid. In addition, elongation at break and modulus also increase similar to tensile strength at that same ratio. The author claimed that the hybridization of bamboo fiber over kenaf fiber has improved the mechanical properties of hybrid composites. This phenomenon occurs because bamboo fiber has good mechanical properties compared to kenaf [134]. The increase in elongation at the break on bamboo increased compared to kenaf, thus also increasing the stretch level of hybrid composites. The findings are supported by Zweben [141] and Thiagamani et al. [142], and the combination of high and low elongation at fractures in the polymer composites is supported by increasing the level of stretching which then acts as a crack inhibitor on the micromechanical level. The thermal properties of bamboo and natural–synthetic fiber have caused behavioral changes in the thermal decomposition of hydride composites. Thermal properties of the bamboo hybrid composites are also performed to determine the stability of the hybrid composite. This test can also determine its thermal stability. The decomposition temperature of hybrid composites depends on the value of each material used. Increasing the percentage of content in composites will also affect the temperature, the maximum temperature, and the final degradation temperature where the temperature will increase due to the lignin content in the material, especially in natural materials [143,144]. Chee et al. [144] studied the thermal properties of bamboo- and kenaf-reinforced epoxy hybrid composites with different fiber loading. The thermal properties tests such as TGA and DSC have been used to analyze the properties of hybrid composites. From the TGA results, the graph trends are similar to those of thermoplastic and thermoset composites. There are four phases involved, i.e., loss of moisture content, degradation of hemicellulose, degradation of cellulose/lignin, and residual ash. From TGA results, the bamboo with a high content of 70 wt.% and 30 wt.% kenaf have the highest thermal stability compared with other ratios. This was followed by DMA results, whereby increasing the bamboo content in hybrid composites also improves the performance of thermal properties. The author concluded that the use of natural fiber, especially bamboo, has a positive impact on thermal properties and argues that bamboo has a high potential to be fabricated and applied to the external use of building materials, such as roofing, siding, and railing. In addition, the effects such as microfiber retraction, fiber surface damage, and fiber exposure due to damage to the polymer matrix can cause negative effects on mechanical and other properties. In addition, the effect of moisture is also one of the weak factors as it results in swelling and micro-cracks hybrid composites.

Therefore, the improvement in the chemical treatment for thermal stability depends on the temperature and immersion time, the type of treatment, and chemical concentration. The immersion time and excessive concentration have also affected the surface of the fibers and then affect the thermal and mechanical properties.

## 3. Economic Value, Challenges, and Future Perspective for Bamboo-Based Composites

Bamboo fiber has a very high commercial value in a variety of industries, from upstream to downstream. Bamboo fiber is forecasted to achieve a global market of USD 98.30 billion by 2025 [145]. It is a versatile feedstock for industrial products and furniture, which explains why it is such high demand within these industries. Thus, the bamboo industry growth will subsequently result in an improvement in the socioeconomic status of the community, hence minimizing or eliminating deprivation, widespread poverty, and underdevelopment in local societies.

**Figure 4:**
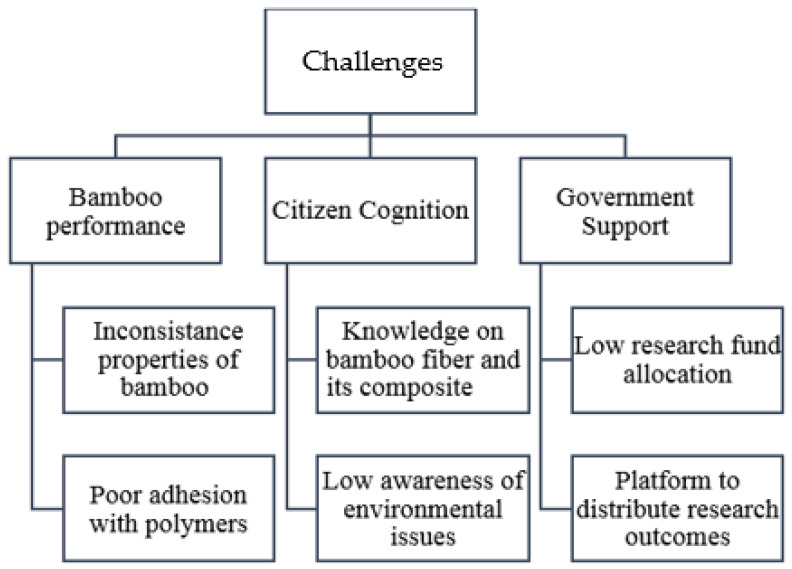
Challenges faced by bamboo composites worldwide.

A balanced supply–demand environment is predicted to result in a price increase for bamboo fiber. Unfortunately, bamboo-fiber-reinforced polymer composites appear to be of no commercial value at this stage. The bamboo fiber reinforcement is compatible with the polymer composites of synthetic fiber. Even so, several obstacles must be overcome before bamboo-reinforced polymer composites can be widely applied. The global challenges of the development of bamboo-fiber-reinforced polymer composites are summarized in Figure 4.

Nearly identical to other natural fibers, the properties of bamboo fiber are primarily determined by the chemical components of the fiber. Nevertheless, high inconsistency degrees in the chemical components of individual bamboo fibers were discovered, indicating that the properties vary between fibers. This has caused manufacturers to surrender the use of bamboo fiber as an alternative to synthetic fibers that are otherwise identical. Besides that, the hydrophilicity of the bamboo fiber is incompatible with hydrophobic polymers. Worse yet, the addition of bamboo fibers increased the water absorption capacity of the material that can accelerate biodegradation, leading to earlier geometrical integrity and functionality failures of the composite. This phenomenon is completely unacceptable for advanced products application, as unexpected malfunctions can result in the loss of large sums of money and/or valuable lives.

In Brazil, between the years 1995 and 2005, many studies in bamboo as reinforcement for concrete were performed [146]. Seven bamboo species were evaluated to determine the most suitable species for use as lightweight concrete beams reinforcement. This study demonstrated that concrete beams reinforced with bamboo had substantial load-bearing capacities compared to unreinforced beams and were stronger than steel-reinforced concrete beams.

Regrettably, the long-term behavior of bamboo in concrete structures has remained a source of contention for numerous researchers. Over time, the natural bamboo exposure to the concrete matrix results in water absorption by the bamboo from the concrete, resulting in swelling of bamboo material. Recurring swelling and shrinkage of natural bamboo caused sudden detaching of the bamboo material from the concrete matrix. This led to a near-complete loss of the reinforced concrete member’s structural load-bearing capacity.

While the majority of studies on surface treatments describe an improvement in the properties of bamboo composites, there is an increase in the overall cost and production cycle time, presenting the industry with a selection dilemma. Luckily, environmental stewardship awareness is increasing nowadays. Bamboo fibers have evolved into a selling point or a gimmick for businesses seeking to improve their reputation. Nonetheless, industrial stakeholders must take the lead and begin utilizing bamboo fiber as reinforcement, as this is the future direction of material development. Collaboration with industry and funding from industry are critical criteria for developing bamboo-reinforced polymer composite products, particularly during the commercialization stage. Industry collaborators’ comments are extremely valuable because they comprehend the consumer’s needs concerning the product.

Regrettably, citizens’ awareness of environmental issues remains inadequate. Globally, waste production has increased dramatically over the years, and there are no indicators of it slowing. By 2050, global municipal solid waste production is predicted to increase by approximately 70% to 3.4 billion metric tons [147]. The plastic waste segment (particularly single-use masks, gloves, and other personal protective equipment (PPE)) is expected to grow significantly from 2020 onwards as a result of the COVID-19 pandemic. Psychologically, people are averse to purchasing products they are unfamiliar with. Thus, increasing citizens’ awareness of bamboo fibers and the fact that bamboo-composite products are highly compatible with advanced applications will help alleviate the world’s saturated municipal solid waste situation. Additionally, global researchers are currently working to develop PPE from natural fibers to reduce reliance on conventional plastic [148]. Researchers could use this opportunity to research disposal masks made of bamboo’s natural fibers.

Now is the ideal time to educate the public about bamboo composites that would require the government and universities to lend their full support. Due to a lack of appropriate platforms for publishing research achievements, innovations remain within the research community and are not shared with the general public. The government should establish visible platforms for spreading research discoveries to all levels of citizens. Newspapers, social media, public campaigns, and/or community activities are all effective means of disseminating research findings of bamboo composites. Another issue is a lack of funding, which slows the progress of research projects and limits enrolment and presenting at international conferences.

Regrettably, research funding is limited globally throughout this COVID-19 pandemic duration. It is comprehensible that governments must prioritize the social economy’s recovery. In the future, advancement should be initiated on a variety of fronts to internationalize bamboo composites. As a result, this review summarizes and shares current knowledge about bamboo-reinforced polymer composites, enabling researchers to refocus their interests and plans for future research on bamboo-reinforced polymer composites.

## 4. Conclusions

The use of natural materials for manufacturing industry applications challenges all researchers to improve appropriate techniques for durability and product quality by using natural materials reinforced with polymer composites. This review suggests that natural materials, especially bamboo, have the potential to be used as a reinforcing material in the polymer matrix. Additionally, this bamboo has a high impact on the environment and new users. The mechanical, physical, and thermal properties of bamboo can have an enormously positive impact on the manufacturing industry. Besides, the use of bamboo material is a renewable material and, in addition, gives the effect of very low technical preparation and raw material costs. The availability of environmentally friendly materials, especially bamboo, can reduce plant waste. Effects of the use of bamboo will have a positive effect on human beings where the opportunity to generate employment and finance, especially for the rural population. More studies and systematic research on the use of bamboo will lead to greater alignment once commercial crops become one of the most important resources in the manufacturing industry.

## Figures and Tables

**Table 1:** Recently review studies in bamboo-based composites.

Year	Reference	Remark	Processing Technique	Water Absorption	Tensile Strength	Flexural Strength	Impact Strength	Thermal Test	Hybrid Composites
2017	Kenan Song, Xiaofeng Ren and Longhe Zhang [32]	Study on mechanical/thermal and characteristics performance	√		√	√		√	√
2017	Calvin Yap Thai Ming, Wong King Jye and Haris Ahmad Israr Ahmad [33]	Study on mechanical performance			√	√	√		
2018	Jan E.G. van Dam, H. Wolter Elbersen and Claudia M. Daza Montaño [34]	Focus on the potential bamboo in biobased economy	√						
2018	Yıldızhan et al. [35]	Study on the mechanical and chemical properties			√				
2018	S. A. H. Roslan, Z. A. Rasid and M. Z. Hassan [36]	Study on fiber extraction and mechanical properties	√		√	√			
2018	Pramudi et al. [37]	Study on parameter and tensile behavior			√				
2022	Jawaid et al. [38]	Investigated on bamboo- and kenaf-reinforced nanocomposites on mechanical and thermal performance		√	√	√			√

**Table 2:** Natural fibers production [43,44].

Fiber	Annual Production (10^3^ Tons)	Origin
Abaca	70	Leaf
Bamboo	10,000	Stem
Banana	200	Stem
Coir	100	Fruit
Cotton Lint	18,500	Stem
Flax	810	Stem
Hemp	215	Stem
Jute	2500	Stem
Kenaf	770	Stem
Ramie	100	Stem
Rice straw	Abundant	Fruit/grain
Wood	1,750,000	Stem

**Table 3:** Chemical composition of natural fibers [58,59,60].

Fibers	Cellulose (wt.%)	Hemicellulose (wt.%)	Lignin (wt.%)	Waxes (wt.%)
Abaca	56–63	20–25	12–131	-
Bagasse	55.2	16.8	25.3	-
Bamboo	26–43	30	21–31	-
Banana	63–64	-	5–11	-
Coir	32–43	0.15–0.25	40–45	-
Cotton	85–90	5.7	-	0.6
Curaua	7.36	9.9	7.5	-
Flax	71	18.6–20.6	2.2–20.6	1.5–1.7
Hemp	68–74	15–22.4	3.5–10	0.8
Jute	61–71.5	13.6–20.4	12–13	0.5
Kenaf	45–72	20.3–21.5	8–13	-
Pineapple	80.5	17.5	8.3	-
Ramie	68.6–76.2	13–16	0.6–0.7	0.3
Sisal	65–78	10–14	9.9–14	2

**Table 4:** Natural and E-glass fibers properties [61,62].

Fibers	Tensile (MPa)	Young’s Modulus (GPa)	Elongation at Break (%)	Density (g/cm^3^)
Abaca	400	12	3–10	1.5
Bagasse	350	22	5.8	0.89
Bamboo	290	17	-	1.25
Banana	529–914	27–32	5.9	1.35
Coir	220	6	15–25	1.25
Cotton	400	11.8	3–10	1.51
Curaua	550–1150	11.8	3.7–4.3	1.4
Flax	800–1500	60–80	1.2–1.6	1.4
Hemp	550–900	70	1.6	1.48
Jute	410–780	26.5	1.9	1.48
Kenaf	930	53	1.6	-
Pineapple	413–1627	60–82	14.5	1.44
Ramie	500	44	2	1.5
Sisal	610–720	2–3	2–3	1.34
E-glass	2400	73	3	2.55

**Table 5:** Mechanical properties of bamboo species [70].

Bamboo Species	Modulus of Rupture (MPa)	Modulus Elasticity (MPa)	Shear Strength (MPa)	Compression Strength (MPa)
*Bambusa blumeana*	99.8	4100	4.5	24
*Bambusa vulgaris*	62.3	6100	4	25.3
*Balanocarpus levis*	122	1800	13.7	69
*Dendrocalamus asper*	85.7	6300	5.4	31.5
*Gigantochloa scortechinii*	52.4	4800	4.3	27
*Gigantochloa levis*	78.5	5100	4.8	40
*Koompasia malaccensis*	100	1700	10	54.7

**Table 6:** Bamboo-reinforced polymer thermoplastic composites and testing method.

Bamboo (Type)	Polymer Thermoplastic	Manufacturing Methods	Applied Testing Method	Ref.
Flour	High-density polyethylene (HDPE)	Twin screw extruders and injection molding	Static mechanical test, dynamic mechanical analyzer (DM), scanning electron microscope (SEM)	[78]
Fiber	Polypropylene (PP)	Twin screw extruders and injection molding	Tensile strength, flexural strength, impact strength, water absorption, and thermogravimetric analysis (TGA)	[82]
Fiber	PP	Hot press	Tensile strength, SEM, and steam explosion technique	[83]
Fiber	HDPE	Melt blending and hot press	Mechanical test and SEM	[84]
Fiber	Polyvinyl chloride (PVC)	Hot–cool mixer, twin screw extruders, and hot press	Mechanical test	[72]
Fiber	Polyethylene (PE)	Twin screw extruders	Mechanical test and thermal properties	[85]
Fiber	PP	Two roll mill and hot press	Mechanical test, physical and SEM	[86]

**Table 7:** Mechanical and physical properties of bamboo-reinforced thermoplastic polymer.

Thermoplastic Matrix	Tensile Strength (MPa)	Flexural Strength (MPa)	Impact Strength (kJ/m)	Water Absorption (%)	Ref.
HDPE	19–44.7	20–36	2.4–4.9	1.5–10	[84,85,93,94]
LDPE	9.2–9.5	17.57	8.35	-	[95,96]
PP	25.5–63	38.8–80	2.94–3.13	2.07–3.76	[31,82,86,97]
Polystrene	25–69	27–29	1.14	2.79	[98,99]
PLA	26–41.4	85	6	-	[100,101,102]

**Table 8:** TGA results for bamboo–HDPE composites and neat HDPE [85] (modified).

Samples	T1_on_ (°C)	T_max_ (°C)	Residue at 600 °C (%)
1	341.36	466.48	2.19
2	339.37	474.55	2.58
3	321.03	473.55	3.02
4	304.13	472.53	3.6
5	297.71	470.51	4.01
6	277.53	468.45	16.43
Neat HDPE	427.64	-	0.49

**Table 9:** Thermoset Properties.

Thermoset	Density (g/cm^3^)	Tensile Strength (MPa)	Young Modulus (GPa)	Elongation (%)	Ref.
Epoxy	1.1–1.4	35–90	2.1–6	1.9–3.5	[104,106,107,108]
Phenolic	1.3	55–55	2.7–4.1	-	[104]
Polyester	1.2–1.5	61–63	1–4	2.5–4.7	[97,98,101]
Polyimides	1.46	120	3.5–4.5	-	[104]
Vinyl Ester	1.2–1.4	80–120	2.9–11.9	3–5	[104,107]

**Table 10:** Common bamboo-reinforced thermoset polymer composites from the literature.

Bamboo Type	Polymer Thermoset	Manufacturing Methods	Applied Testing Methods	Ref.
Fiber	Epoxy	Resin transfer molding (RTM)	Tensile and flexural test, and hydrothermal ageing test	[109]
Fiber	Epoxy	Hand layout	Mechanical test	[110]
Fiber	Unsaturated polyester	Hand layout	Mechanical test	[111]
Fiber	Epoxy	Hand layout	Flexural, quasi-static fracture toughness, thermal and FTIR test	[112]
Powder	Epoxy	Hand layout	Thermal test	[113]
Fiber	Polyester	Hand layout	Mechanical test	[114]
Solid	Phenol–formaldehyde	Impregnation	Dynamic mechanical test (DMA), compression test, measurement of friction coefficient, and differential scanning calorimetry (DSC)	[115]
Strips	Phenol–formaldehyde (PF)	Winding	Compression test	[116]
Fiber	Polyester	Vacuum infusion	Fracture and tensile test	[117]

**Table 11:** Mechanical and physical properties of bamboo-reinforced thermoset polymer.

Thermoset Matrix	Tensile Strength (MPa)	Flexural Strength (MPa)	Impact Strength (kJ/m)	Water Absorption (%)	Ref.
Epoxy	142.86–291.67	141.39–182.29	-	19	[109,112,121]
Phenolic	114.4–354.78	-	-	7.98	[122]
Polyester	98.4–191.3	50–128.5	5–20	4–12.05	[111,117,123,124]
Vinyl Ester	48.06–119.39	106.81–161.58	-	6–14	[125]

**Table 12:** Bamboo-reinforced polymer hybrid composites and preparation methods.

Hybrid	Resin	Method	Ref.
Date palm	Epoxy	Hand layout technique	[131]
Jute	Low-density polyethylene (LDPE)	Hotpress technique	[132]
Jute	Vinyl ester	Hand layout technique	[133]
Kenaf	Epoxy	Hand layout technique	[134]
Pineapple leaf/coir fiber	Polyester	Hotpress technique	[135]
Sisal	Polyester	Hand layout technique	[136]
Sugarcane bagasse	Polyurethane (PU) foam	Hand layout and compression molding technique	[137]
Carbon nanotubes (CNT)	Epoxy	Hand layout technique	[76]
E-glass	Epoxy	Hand layout technique	[138]
Glass	PP	Hotpress technique	[139]

## Data Availability

No new data were created or analyzed in this study. Data sharing is not applicable to this article.
